# Transforming Growth Factor Beta3 is Required for Cardiovascular Development

**DOI:** 10.3390/jcdd7020019

**Published:** 2020-05-24

**Authors:** Mrinmay Chakrabarti, Nadia Al-Sammarraie, Mengistu G. Gebere, Aniket Bhattacharya, Sunita Chopra, John Johnson, Edsel A. Peña, John F. Eberth, Robert E. Poelmann, Adriana C. Gittenberger-de Groot, Mohamad Azhar

**Affiliations:** 1 Department of Cell Biology and Anatomy, University of South Carolina School of Medicine, Columbia, SC 29209, USA; Mrinmay.Chakrabarti@uscmed.sc.edu (M.C.); Nadia.Al-Sammarraie@uscmed.sc.edu (N.A.-S.); Mengistu.Gebere@uscmed.sc.edu (M.G.G.); Aniket.Bhattacharya@uscmed.sc.edu (A.B.); sunita.chopra42@gmail.com (S.C.); John.Johnson@uscmed.sc.edu (J.J.); John.Eberth@uscmed.sc.edu (J.F.E.); 2 Department of Statistics, University of South Carolina, Columbia, SC 290208, USA; pena@stat.sc.edu; 3 Department of Cardiology, Leiden University Medical Center, 2333 ZC Leiden, The Netherlands; R.E.Poelmann@lumc.nl (R.E.P.); A.C.Gittenberger-de_Groot@lumc.nl (A.C.G.-d.G.); 4 William Jennings Bryan Dorn VA Medical Center, Columbia, SC 29209, USA

**Keywords:** transforming growth factor beta-3, cardiac development, loeys dietz syndrome-5, arrhythmogenic right ventricular dysplasia, rienhoff syndrome, cleft palate, congenital heart disease, outflow tract septation, signaling networks

## Abstract

Transforming growth factor beta3 (*TGFB3*) gene mutations in patients of arrhythmogenic right ventricular dysplasia/cardiomyopathy (ARVD1) and Loeys-Dietz syndrome-5 (LDS5)/Rienhoff syndrome are associated with cardiomyopathy, cardiac arrhythmia, cardiac fibrosis, cleft palate, aortic aneurysms, and valvular heart disease. Although the developing heart of embryos express *Tgfb3*, its overarching role remains unclear in cardiovascular development and disease. We used histological, immunohistochemical, and molecular analyses of *Tgfb3*^−/−^ fetuses and compared them to wildtype littermate controls. The cardiovascular phenotypes were diverse with approximately two thirds of the *Tgfb3*^−/−^ fetuses having one or more cardiovascular malformations, including abnormal ventricular myocardium (particularly of the right ventricle), outflow tract septal and alignment defects, abnormal aortic and pulmonary trunk walls, and thickening of semilunar and/or atrioventricular valves. Ventricular septal defects (VSD) including the perimembranous VSDs were observed in *Tgfb3*^−/−^ fetuses with myocardial defects often accompanied by the muscular type VSD. In vitro studies using TGFβ3-deficient fibroblasts in 3-D collagen lattice formation assays indicated that TGFβ3 was required for collagen matrix reorganization. Biochemical studies indicated the ‘paradoxically’ increased activation of canonical (SMAD-dependent) and noncanonical (MAP kinase-dependent) pathways. TGFβ3 is required for cardiovascular development to maintain a balance of canonical and noncanonical TGFβ signaling pathways.

## 1. Introduction

Transforming growth factor beta3 (TGFβ3) is a multifunctional growth factor and cytokine well-known for its involvement in craniofacial development [1,2,3,4,5]. In humans, the TGFB3 gene is involved in arrhythmogenic right ventricular dysplasia (ARVD) familial 1 (ARVD1) (OMIM#107970) [6,7], now being referred to as arrhythmogenic right ventricular cardiomyopathy (ARVC). ARVC involves progressive fibrofatty replacement of the myocardium, resulting in ventricular tachycardia and sudden death in young athletes and patients. The ARVC primarily affects the right ventricle (RV) but emerging evidence suggests that it may also affect the left too (LV) [6,8]. Overall, presentation of the disease is highly variable among affected individuals. Furthermore, *TGFB3* mutations in Loeys-Dietz Syndrome 5 (LDS5)/Rienhoff syndrome (RNHF) (OMIM#615582) are associated with connective tissue disorders and Marfan syndrome (MFS)-like features, including congenital heart defects (CHD), aortic aneurysms, and valvular disease [9,10,11]. For instance, aorta from LDS5 patients exhibit paradoxically increased TGFβ signaling, which is thought to be involved in the diverse aortopathies [9]. Other features of these patients overlap clinically with Shprintzen–Goldberg syndrome (SGS) (OMIM#182212) and Marfan syndrome (MFS) (OMIM#154700), including cleft palate. De novo *TGFB3* mutations also cause congenital syndromes characterized by a combination of clinical features of Beals-Hecht syndrome (CCA) (OMIM#121050), RNHF, MFS, and Loeys-Dietz syndrome (LDS) [12,13,14].

TGFβ3 has been implicated in several cellular processes in a context-dependent and tissue-specific fashion, including epithelial-mesenchymal transition (EMT), cell growth, apoptosis, differentiation, extracellular matrix (ECM) production and remodelling, all of which are critical processes involved in both the development and homeostasis of cardiovascular tissues [15,16,17,18,19]. TGFβ3 interacts with the heteromeric TGFβR2 and TGFβR1 receptor complex, which results in the phosphorylation and activation of SMAD2 and SMAD3 [20]. In association with SMAD4, the phosphorylated SMAD2/3 molecules migrate to the nucleus [21]. TGFβ ligands can induce SMAD1/5/9, so-called BMP-driven SMADs, via involvement of BMP Type I receptors in endothelial cells and/or fibroblasts, thereby bringing about crosstalk between TGFβ and BMP signaling pathways [22]. In addition, TGFβ ligands can signal through non-SMAD mechanisms, including MAP kinase pathways [23]. Thus, given the non-overlapping, dozens of phenotypes among the TGFβ3 ligand knockout mice [24], as well as the multiple TGFβ signaling pathways that have been identified [25], there are several potential mechanisms through which TGFβ3 can affect the complex differentiation and morphogenetic processes required to develop the essential components of the heart.

The expression of *TGFB3* has been detected in the developing mouse heart and adult human heart [26,27]. TGFβ signaling is a critical contributor to collagen accumulation and defective collagen reorganization during fibrofatty lesion formation in ARVD1 and myocardial fibrosis in the infarcted failing hearts [28]. To address the role of TGFβ3 in vivo, two different laboratories independently generated *Tgfb3* knockout (*Tgfb3*^−/−^) mice [1,2]. Unfortunately, *Tgfb3*^−/−^ newborn mice have a cleft palate and die within 24 h of birth. Although cardiovascular disease was not the focus of the two studies, none of the studies reported any congenital heart defects in the *Tgfb3*^−/−^ fetuses. Consequently, the exact role of TGFβ3 in cardiovascular development remains unknown. Given the recent reports of genetic involvement of TGFB3 mutations in the cardiovascular disease in humans [6,7,9,12], our paradigm-shifting findings demonstrate that the majority of *Tgfb3*^−/−^ fetuses develop both cardiac outflow tract (OFT) and atrioventricular (AV) canal defects and that the loss of *Tgfb3* leads to major myocardial defects, particularly affecting the right ventricular myocardium. Our data also identify downstream mechanisms and specific components of the SMAD- and MAP kinase (MAPK)-dependent signaling pathways that are involved in cardiovascular malformations in TGFβ3-deficient mice.

## 2. Materials and Methods

### 2.1. Ethics Statement

All animal procedures were performed according to the Guidelines for the Care and Use of Laboratory Animals published by the National Institutes of Health and were approved by the Institutional Animal Care and Use Committee of the University of South Carolina (Animal use proposal reference #: 2451-101423-042519, protocol approval date: 25 April 2019, protocol expiration date: 25 April 2022) Mice were euthanized by an overdose of isoflurane in a sealed container as approved by the IACUC.

### 2.2. Mouse Strains

Mice were housed at the University of South Carolina Animal Research Facility at the School of Medicine. The Institutional Animal Care and Use Committee (University of South Carolina) approved all mouse breeding and experimental procedures. *Tgfb3*^+/−^ mice [1] were backcrossed on to a C57BL/6 background for more than 12 generations, and maintained on the C57BL/6 genetic background. *Tgfb3*^−/−^ embryos were generated and genotyped as described [1]. Timed mating was performed, and the noontime of the positive vaginal plugs was considered as embryonic day (E) 0.5. The DNA sequence of the *Tgfb3* specific primers that were used for genotyping includes: TGGGAGTCATGGCTGTAACT (IMF-10, forward primer), CACTCACACTGGCAAGTAGT (IMR-10, reverse primer). PCR genotyping was done as described [1]. PCR genotyping of embryonic tissues genomic DNA identified wildtype, *Tgfb3*^+/−^, and *Tgfb3*^−/−^ fetuses for experiments.

### 2.3. Histology and Immunohistochemistry

Wildtype, *Tgfb3*^+/−^, and *Tgfb3*^−/−^ embryos/fetuses were collected between E13.5 and E18.5 and processed for histological and immunohistochemical, morphometric, and molecular analyses. Hematoxylin and eosin (H&E) staining was performed on 7 μm thick serial sections of the heart for routine histological examination. Immunohistochemistry (IHC) was done on 4% paraformaldehyde fixed 7 μm thick paraffin sections by using immunostaining kits (LSAB2 kit, Universal, HRP Rabbit/Mouse (Cat #: K0675), according to the protocol of the manufacturer (Agilent Dako, USA), and as previously published [29]. Antigen retrieval was performed in Target Retrieval Solution pH 6.0 (catalog no. S1700; Agilent Dako, USA) for 15–20 min at 95°C. Anti-muscle actin (Clone: HHF35, catalog no. M0635; Agilent Dako; 1:50 dilution) immunostained sections were counterstained with hematoxylin nuclear stain. All sections were visualized and photographed under brightfield optics on a Nikon Optiphot-2 (equipped with AxioCam MRc Camera) and EVOS TM FL Auto Imaging System (ThermoFisher, Inc, Grand Island, NY, USA). For photographing elastin autofluorescence in hematoxylin and eosin stained sections, 470/22 excitation and 510/42 emission filters were used on a Zeiss Axiovert 200 (Thornwood, NY, USA) (equipped with Axiocam 503 color camera and ZEN 2.3 SP1 Imaging software).

### 2.4. TUNEL Staining

The TUNEL assay was used to detect apoptosis in outflow tract cushions. Paraffin sections from E13.5-14.5 OFT cushions from wildtype and *Tgfb3*^−/−^ embryos were subjected to the TUNEL assay using FragEL™ DNA Fragmentation Detection Kit, Colorimetric - TdT Enzyme (Sigma-Aldrich, Cat#QIA33, St. Louis, MO, USA). All nuclei were also stained with methyl green (Vector Laboratories). Stained samples were observed with a Nikon E400 light microscope and photographs (10 × Obj) were used for quantitative analysis (Image Pro Plus software, Media Cybernetics, Rockville, MD, USA) by analyzing up to 500 cells/sample from 4–5 sections of the OFT septum spaced 24 µm apart for 3 samples each of *Tgfb3*^−/−^ and control embryos. The percent (%) average apoptosis from each sample was plotted using a scatter plot.

### 2.5. Cell Proliferation

Cell proliferation was monitored by immunohistochemistry. Paraffin sections of E13.5-14.5 outflow tract septal cushions from *Tgfb3*^−/−^ and wildtype embryos were subjected to immunohistochemistry with a phospho-histone H3 (Ser10) antibody (Cell Signaling Inc, #9701, Danvers, MA, USA). Stained samples were observed with a Nikon E400 light microscope and photographs (×Obj) were used for quantitative analysis (Image Pro Plus software) by analyzing the number of pHH3-positive cells per sample collected from the region around fibrous OFT septum in 4–5 sections spaced 24 µm apart for 3 samples each of *Tgfb3*^−/−^ and control embryos. 

### 2.6. AMIRA Three-Dimensional (3-D) Reconstruction and Volumetric Analysis

Seven-micrometer paraffin serial sections were obtained on a microtome and stained with hematoxylin and eosin (H&E). TIFF images of the complete aortic valves from wildtype and *Tgfb3*^−/−^ embryos (E4.5-15.5) were captured using the 4× objective on a Nikon Optiphot-2 light microscope and loaded into the AMIRA software package (FEI Visualization Science Group, Burlington, MA, USA) [30]. All sections were aligned, and the aortic valve (left and right coronary and non-coronary leaflets) was segmented. A 3D model of the aortic valves was then generated from which the volume of the aortic valve cushion was determined for both wildtype and *Tgfb3*^−/−^ fetuses. Results were compared using a Student’s *t*-test.

### 2.7. RNAscope In Situ Hybridization

In situ hybridization was carried out using a *Tgfb3* RNA probe synthesized by Advanced Cell Diagnostics (Newark, CA, USA) (ACD, #406211) [31]. Detection was done using the RNAscope 2.5 HD Duplex Reagent Mouse Kit (Cat. No 322430) according to the manufacturer’s protocol (ACD). After the signal had developed, sections dehydrated in a series of ethanol and xylene were mounted using permount (Vector Lab, Burlingame, CA, USA).

### 2.8. Collagen Gel Contraction Assays

Three independent mouse embryonic fibroblast lines were generated for each wildtype and *Tgfb3* knock out E14.5 embryos as described [32]. Mouse fibroblasts were maintained in DMEM (Invitrogen) supplemented with 10% bovine serum, 5% fetal calf serum and 1% penicillin/streptomycin (Sigma-Aldrich, St. Louis, MO, USA) at 37 °C/5% CO2. In addition to reorganization, collagen contraction assays are indicative of the capacity for embedded cells to generate mechanical loads [33]. The capability of mouse fibroblasts to form lattices in collagen gels was assessed by plating 105 cells in 2mg/mL collagen type-I (in 18mM acetic acid) prepared in complete media and supplemented with 0.1 M NaOH, as detailed [34]. Free-floating collagen gels were incubated at 37 °C for 5 days, with or without recombinant TGFβ1 (0.1 ng/mL or 1 ng/mL, Sigma-Aldrich, St. Louis, MO). Images were acquired and best-fit shapes (circle or free form) were used to calculate the surface area of the gels using image processing utilities in Zeiss (ZEN) Imaging Software. The initial area of the collagen gel on day 1 was used to normalize across experiments. The percent (%) decrease in gel surface area after 5 days denotes the degree of collagen contraction, with higher values indicate greater contraction [35]. The results are presented as scatter dot-plots with the box denoting the mean ± SEM and dots representing individual data points.

### 2.9. Western Blot Analysis

Western blotting was performed with the samples of individual heart with the great vessels of the arterial pole, which were collected from wild type and *Tgfb3* knockout (E18.5) fetuses. Since there was not enough protein available for Western blot analyses from individual heart at mid-gestation (E14.5), we used pooled samples (three fetal hearts and aortas/sample) from wildtype, *Tgfb3*^+/−^ and *Tgfb3*^−/−^ fetuses at E14.5. The fetal hearts were cut into small pieces and homogenized using Wheaton tapered tissue grinders (Thermo Scientific, Rockford, IL) in M-PER mammalian protein extraction reagent (Thermo Scientific, Rockford, IL) with complete mini protease inhibitor cocktail (Sigma-Aldrich, states. Louis, MO, USA) and Halt protease and phosphatase inhibitor single-use cocktail (Thermo Scientific, Rockford, IL, USA) as per the manufacturer’s protocol. Homogenized tissue lysates were subjected to brief sonication for 20 s on ice and kept at room temperature for 20 min. Then, centrifugation was performed at 15,000 rpm for 20 min at 4 °C and the supernatants were collected. Total protein concentration in the supernatant was determined using Pierce BCA protein assay kit (Thermo Scientific, Rockford, IL, USA) and the samples were stored at −80 °C until further use. For pooled samples, we collected and pooled three hearts and ascending aortas per sample after genotyping from wildtype, *Tgfb3*^+/−^ and *Tgfb3*^−/−^ fetuses (E14.5). Western blotting was performed with equal amounts of protein samples and the primary IgG antibodies against phospho-SMAD2 (Cell Signaling Technology, cat #3108), SMAD2 (Cell Signaling, cat #5339), phospho-SMAD3 (Cell Signaling, cat #9520), SMAD3 (Cell Signaling, cat #9523), phospho-SMAD1/5 (Cell Signaling, cat #9516), SMAD1/5 (Cell Signaling, cat #9743), phospho-p38 (Cell Signaling, cat #4511), p38 (Cell Signaling, cat #8690), phospho-ERK1/2(Cell Signaling, cat #4370), ERK1/2 (Cell Signaling, cat #4695) at a dilution of 1:1000. Primary IgG antibodies against all these proteins were purchased from Cell Signaling Technology, Inc. (Danvers, MA, USA). The horseradish peroxidase-conjugated anti-mouse or anti-rabbit secondary IgG antibody (Cell Signaling, cat#7074) was used at 1:5000 dilution to detect a primary IgG antibody. In a separate Western blot, the levels of β-actin in all wildtype and *Tgfb3*^−/−^ tissue samples were determined. Western blots were incubated with Clarity Western ECL detection reagents (Bio-Rad Laboratories, USA) and exposed to X-OMAT AR films (Eastman Kodak, Rochester, NY, USA) for autoradiography. The autoradiograms were scanned on an EPSON scanner using Photoshop software (Adobe Systems, Seattle, WA, USA). β-actin, clone AC-15 monoclonal primary antibody (Sigma-Aldrich, St. Louis, MO, USA) was used as an internal housekeeping control to compare equal loading in the SDS-PAGE. Since the β-actin values were not different between the wildtype and *Tgfb3*^−/−^ tissue samples, the data from all blots were normalized to β-actin values obtained from an independent blot. The ratio of both phosphorylated protein/β-actin and phosphorylated protein/total protein were plotted as scatter dot-plots with the box denoting the mean ± SEM and dots representing individual data points.

Microsoft Excel was used for recording and managing the raw data. Data were presented as mean ± SEM. Continuous data were presented as bar/scatter/dot plots, showing the individual data points together with the average/error bars. All data points regardless whether they were included or excluded from statistical analysis presented on scatter plots. Statistical significance was calculated using either the Student’s *t* test or the Mann–Whitney (nonparametric test) (two-tailed, for two-group comparison) using the GraphPad Prism 8 statistical program (GraphPad, San Diego, CA, USA). Exact p-values were calculated, and the probability values <0.05 were considered as significant.

## 3. Results

### 3.1. Systemic Tgfb3 Deletion Disrupts Cardiac Development

**Figure 1:**
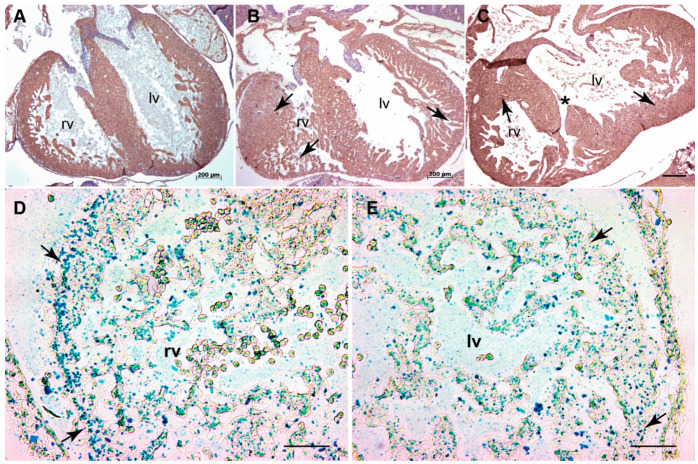
*Tgfb3* deletion leads to an impaired myocardium in mouse embryos. (**A**–**C**), Cross- sections of E15.5-16.5 fetuses showing cardiac muscle actin (clone HHF35) immunohistochemistry for wildtype (**A**) and two different TGFβ3-deficient fetuses (**B**,**C**). TGFβ3-deficient fetuses developed non-uniform right ventricular myocardium with heterogeneous regions of thicker (**B**, left arrow) and thinner (**B**, bottom arrow) myocardium and spongy (**B**, right arrow) and thicker (**C**, right arrow) left ventricular myocardium compared to the wildtype littermate fetus (**A**), Other mutant fetus had a muscular ventricular septal defect (VSD) (**C**, asterisk) (*n* = 6). (**D**,**E**), Representative images revealed *Tgfb3* expression (green-blue punctate dots, arrows) in the compact and trabecular myocardium of the right (**D**) and left ventricles (**E**) from wildtype E12.5 embryo. Scale bars = 200 µm for (**A**–**C**), 50 µm for (**D**,**E**). Abbreviations: rv, right ventricle; lv, left ventricle.

Analysis of hematoxylin and eosin (H&E)-stained serial sections as well as muscle actin immunohistochemistry (via an anti-HHF-35 clone) of selected sections from embryonic day 13.5 to 18.5 was used to determine the cardiac pathomorphology in *Tgfb3*^−/−^ fetuses. Cardiovascular morphology was compared between wildtype (*n* = 14) and *Tgfb3*^−/−^ (*n* = 19) and the cardiovascular malformations in the *Tgfb3*^−/−^ were determined and cataloged. Approximately two thirds cases of the *Tgfb3*^−/−^ fetuses developed malformations of the outflow tract and AV canal (Table 1 and Table S1). Importantly, the data revealed an abnormally smaller right ventricle than the left ventricle and that right ventricular myocardial development was impaired in about 21% cases of the *Tgfb3*^−/−^ fetuses (Figure 1A–C and Figure S1A–C). Approximately 26% of the *Tgfb3*^−/−^ fetuses showed the myocardial defects affecting both right and left ventricles, affecting both the compact and the trabeculated myocardium (Figure 1 and Figure S1). The myocardium of the interventricular septum and apex region was very spongy and poorly formed (Figure 1 A,C and Figure S1A,C). Severely impaired myocardium of the interventricular septum was associated with the muscular type of the ventricular septal defect (VSD) in the *Tgfb3*^−/−^ fetuses (Figure 1A,C and Figure S1D,E). Histological analysis also revealed slightly impaired ventricular myocardium in 1/3 cases of *Tgfb3*^+/−^ fetuses (E14.5) (Figure S1F,G). In situ hybridization analysis of wildtype hearts (E12.5) detected higher expression of *Tgfb3* in the compact and trabecular myocardium of the right ventricle and to a lesser degree in the left ventricle (Figure 1D,E). Overall, these results indicate that TGFβ3 is required for ventricular myocardial development and that it plays a more important role in the formation of the right ventricle.

### 3.2. Tgfb3 Deletion Leads to Outflow Tract Cushion Remodeling and Septal Defects

**Figure 2:**
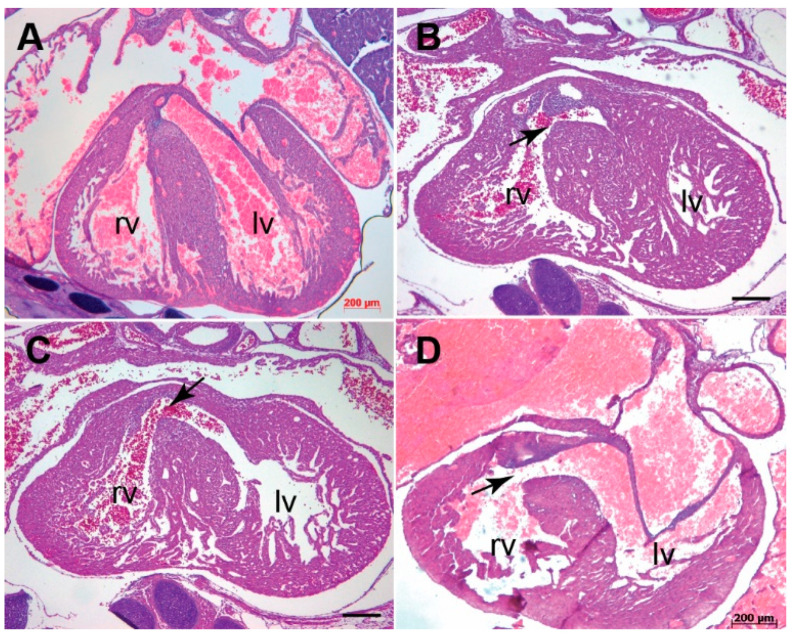
*Tgfb3* knockout mouse fetuses exhibit outflow tract septal and alignment defects. (**A**–**D**), Hematoxylin and eosin staining of hearts for E15.5-16.5 wildtype (**A**) and two different *Tgfb3*^−/−^ fetuses (**B**–**D**). *Tgfb3*^−/−^ fetuses develop both double-outlet right ventricle (**B**, arrow) and perimembranous ventricular septal defect (**C**,**D**; arrows). Scale bars = 200 µm for (**A**–**D**). Abbreviations: rv, right ventricle; lv, left ventricle.

**Figure 3:**
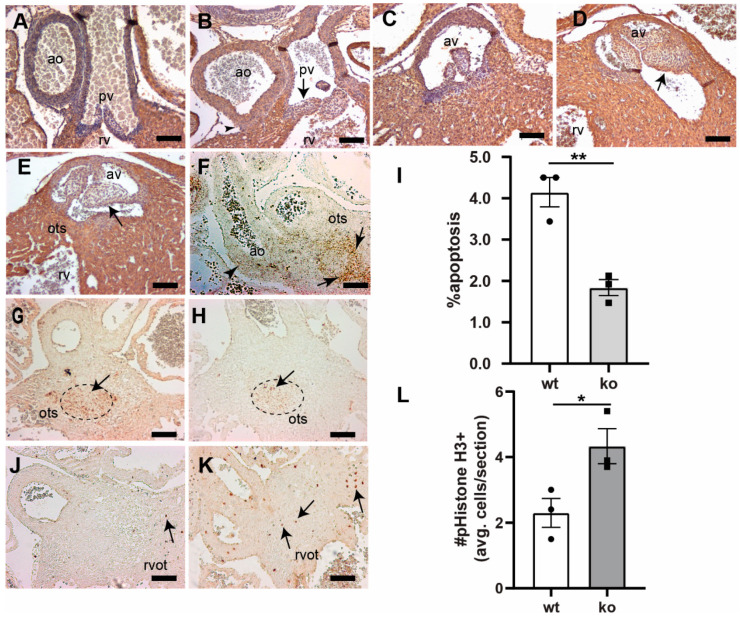
Loss of *Tgfb3* in mice results in the outflow tract cushion and vascular wall defects. (**A**–**E**), Cross-sections of E15.5–16.5 fetuses showing cardiac muscle actin (clone HHF35) immunohistochemistry for wildtype controls (**A**,**C**) and TGFβ3-deficient fetuses (**B**,**D**,**E**). *Tgfb3*^−/−^ fetuses develop dysmorphic pulmonary (**B**) and aortic valve (**D**,**E**), and abnormal ascending aortic and pulmonary trunk walls (**B**) morphology. *Tgfb3*^−/−^ fetuses also demonstrating hypoplastic outlet septum (**E**). (**F**) Representative image of a wildtype embryo (E11.5) using RNAscope in situ hybridization reveals *Tgfb3* expression (brown punctate dots) in the vascular wall (arrowhead) and outflow tract septum (arrow). (**G**,**H**), Apoptosis (E13.5) using TUNEL staining (brown colored nuclei) in outflow tract septum mesenchyme. Compared to wildtype controls (**G**), *Tgfb3*^−/−^ OFT septum have a reduced number of apoptotic cells (**H**). (**I**) Quantification of fraction of cells undergoing apoptosis. Mean ± SEM of % average apoptosis from at least 4 sections for each sample was used for comparison. Quantification was predominantly done in the area of outflow tract septum marked by a dotted circle. Reduced apoptosis in *Tgfb3*^−/−^ hearts was noted as compared to wildtype embryos (** *p* = 0.004, Student’s *t* test; *p* = 0.07, Nonparametric (Mann Whitney test)). (**J**–**L**), Cell proliferation (E13.5) using phospho-histone H3 (Ser10) immunohistochemistry. Mean ± SEM of average pHistoneH3+ cells/section from at least 4 sections for each sample was used for comparison. Quantification was mainly restricted to the region around the fibrous outflow tract septum (**L**). Increased cell proliferation in *Tgfb3*^−/−^ hearts (K, arrows) was observed as compared to wildtype (**J**) embryos (* *p* = 0.04, Student’s *t* test; *p* > 0.05, Nonparametric (Mann Whitney test)). Scale bars = 100 µm for (**A**–**E**,**F**–**H**,**J**,**K**). Abbreviations: rv, right ventricle; av, aortic valve; pv, pulmonary valve; ao, ascending aorta; ots, outlet septum, rvot, right ventricular OFT.

Histological examination of the H&E stained serial sections from wildtype and *Tgfb3*^−/−^ fetuses at E13.5-18.5 revealed that about two thirds of the cases of the homozygous mutant fetuses developed various cardiac outflow tract malformations with variable penetrance (Table 1 and Table S1). Cardiac muscle immunohistochemistry (via anti-cardiac muscle actin) was performed to confirm the OFT malformations. Major cardiac OFT malformations included double-outlet right ventricle (DORV) and a large OFT ventricular septal defect (VSD) with extension into inflow (about 5% cases) (Figure 2A–D), thickening of the pulmonary and aortic valve (about two thirds of cases) (Figure 3A–E and Figure S2A–D), abnormal ascending aortic and pulmonary trunk walls (about 16% cases) (Figure 3A,B and Figure S3). The DORV was mainly subaortic in nature and exhibited defective fusion and remodeling of the RV OFT septum (Figure 2A,B and Figure 3C–E). The walls of the aorta and pulmonary trunk appeared morphologically immature and the vascular smooth muscle cells distributed randomly in the vascular walls of *Tgfb3*^−/−^ fetuses compared to their control littermates (Figure 3A,B and Figure S3). In addition, histological analysis also revealed hypoplastic vascular walls of aorta and/or pulmonary trunk and mildly thickened aortic and/or pulmonary valves in one third of cases of *Tgfb3*^+/−^ fetuses (E14.5) (Figure S2A,B,D,E). There These OFT defects were consistent with the significant expression of *Tgfb3* in OFT cushion mesenchyme and walls of aorta and pulmonary trunk (Figure 3F). Morphometric comparison and volume measurements via AMIRA 3D reconstruction of serial sections confirmed the hyperplastic nature of the OFT cushions in *Tgfb3*^−/−^ fetuses compared to the wildtype littermate fetuses (Figure S4). Since the OFT septum was abnormal in *Tgfb3*^−/−^ embryos, TUNEL assay was used to quantify the apoptosis. The OFT septum consists of two cell populations—cardiac neural crest (NCC) and second heart field (SHF)-derived cells. Apoptosis is a phenomenon of NCC [36]. RNAscope in situ hybridization confirmed that *Tgfb3* is significantly expressed in the OFT septum. Apoptosis centrally in the OFT septum (Figure 3G–I) could activate TGFβ3 in the circle of SHF-derived cells around it for their proper differentiation and proliferation (Figure 3J–L). To get the measurements of the difference in apoptosis between wildtype and *Tgfb3*^−/−^ embryos the number of cells (as indicated in Figure 3I) were counted within the central NCC bound area. TUNEL analysis showed that the apoptosis of cushion mesenchymal cells at the OFT septum was significantly decreased in the affected *Tgfb3*^−/−^ fetuses compared to wildtype littermates (Figure 3G–I). Immunohistochemical analysis via anti-phospho-histone H3 (Ser10) revealed an increase in the myocardial cell proliferation surrounding the central fibrous OFT septum and in the right ventricular myocardium (Figure 3J–L). This indicates that TGFβ3 is required for cardiac apoptosis and proliferation, which are important to cushion mesenchymal differentiation and maturation. Collectively, the data indicate that the loss of *Tgfb3* affects cardiac outflow tract cushion remodeling and septal and alignment processes.

### 3.3. Tgfb3 Deletion Leads to AV Cushion Remodeling Defects

**Figure 4:**
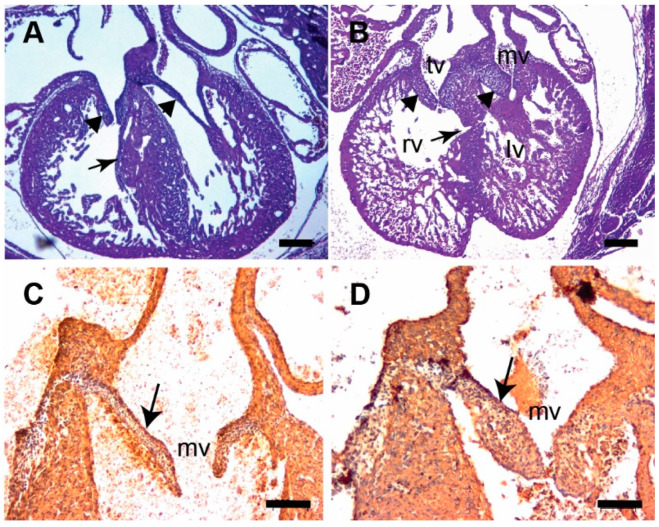
*Tgfb3* knockout fetuses exhibit atrioventricular valve thickening. (**A**,**B**) H&E stained sections of wildtype (**A**) and Tgfb3^−/−^ fetus (E14.5-15.5) showing mitral valve and tricuspid valve thickening (**B**, arrowheads). Note that the ventricular myocardium is thin and abnormal with muscular ventricular septal defect in *Tgfb3*^−/−^ (**B**, arrow). (**C**,**D**), Cardiac muscle actin (clone HHF35) immunohistochemistry of cross-sections of E15.5-16.5 wildtype (**C**) and *Tgfb3*^−/−^ (**D**) fetuses. *Tgfb3*^−/−^ fetus develop thickened mitral valves (**D**, arrow). Scale bars = 200 µm for (**A**,**B**), 50 µm for (**C**,**D**). Abbreviations: rv, right ventricle; lv, left ventricle; tv, tricuspid valve; mv, mitral valve.

Analysis of H&E stained serial sections revealed that approximately 42% cases of *Tgfb3*^−/−^ fetuses developed malformations of the AV valves (Table 1 and Table S1). Mitral and/or tricuspid valve thickening was observed in all affected cases of the overall mutants analyzed (Figure 4A–D). Importantly, a significant proportion of the *Tgfb3*^−/−^ fetuses developed VSD, which included perimembranous VSD (about 21% cases) (Figure 2A,C,D). The perimembranous VSD is normally linked to OFT VSD with an extension into the inlet septum and that it not obligatory related to abnormal AV valves. Thus, the perimembranous VSD is due to abnormal OFT cushion remodeling and is not related to AV cushion remodeling defects. Histological analysis did not reveal the presence of any AV septal defect (AVSD) in *Tgfb3*^−/−^ embryos. These data indicate a requirement of TGFβ3 in the AV cushion remodeling and but not in AV septal formation during heart development.

### 3.4. TGFβ3 Is Required for Extracellular Matrix Reorganization

**Figure 5:**
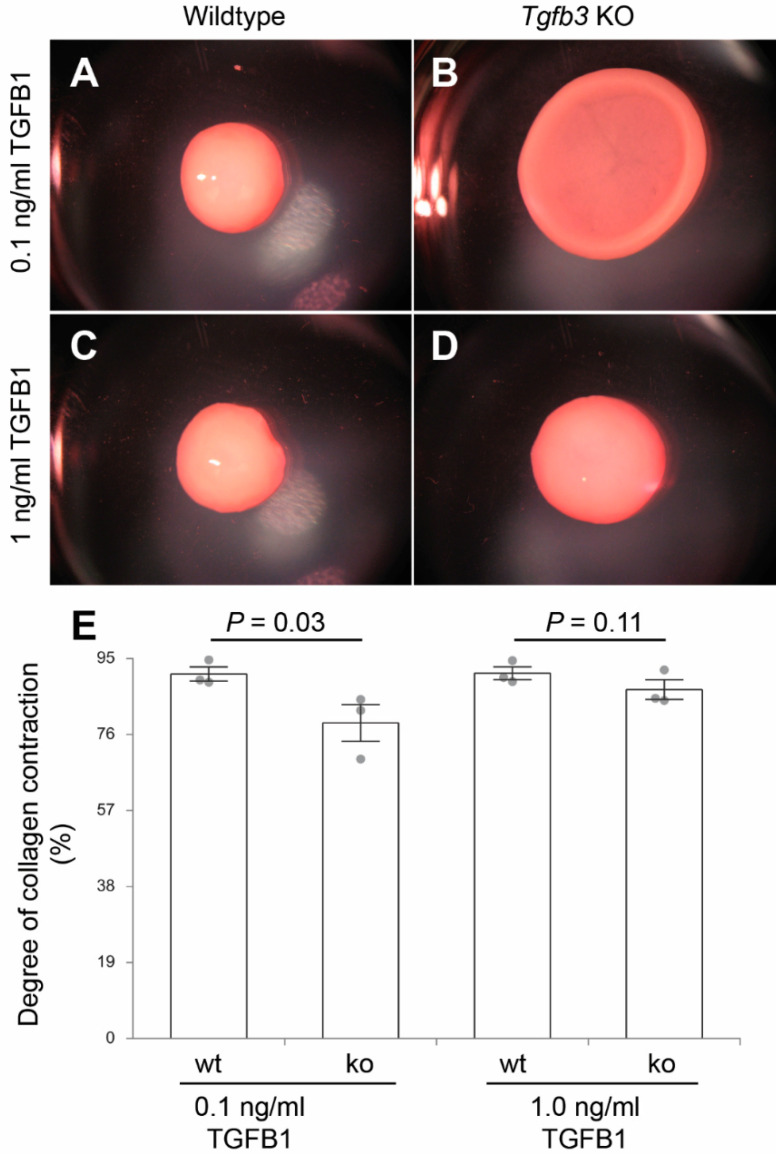
Collagen lattice formation assay. (**A**–**D**), dissecting microscope images of fibroblast-laden collagen gels after 5-days using three independent fibroblast cell lines (*n* = 3) from wildtype (**A**,**C**) or *Tgfb3*^−/−^ embryos (**B**,**D**) in the presence of low (0.1 ng/mL) (**A**,**B**) and high (1 ng/mL) (**C**,**D**) doses of exogenous recombinant TGFB1. TGFβ3-deficient whole mouse embryonic fibroblasts treated with the low dose of exogenous TGFB1 have decreased contractility compared to the wildtype fibroblasts while higher doses rescue contractility and lattice formation (**E**). *p*-Values are indicated in the histogram. Numerical data are presented as scatter dot-plots with the box denoting the mean; error bars identify the SEM.

Given the involvement of TGFB3 in the development of fibrofatty lesions in the ARVD1, TGFβ3-deficient whole mouse embryonic fibroblasts were generated and tested for their ability to reorganize collagen in vitro in a three-dimensional collagen lattice formation assay. TGFβ3-deficient and wildtype fibroblasts were seeded in collagen gels and cultured for five days with or without 0.1 ng/mL and 1 ng/mL TGFβ1. The degree of collagen contraction (i.e., decrease in the surface area of the collagen gel) over time was determined. The wild type fibroblasts contracted in response to exogenously added TGFβ1, as expected, in a dose-dependent fashion. TGFβ3-deficient fibroblasts exhibited a reduced contraction compared to the wild type. Nonetheless, they remained responsive to TGFβ1 induction (Figure 5A–E). The difference in the degree of contraction between TGFβ3-deficient and wildtype whole mouse embryonic fibroblasts was rescued by exogenously added TGFβ1, indicating that the excess TGFβ1 is able to rescue the impaired ability of TGFβ3-deficient whole mouse embryonic fibroblasts to reorganize collagen lattices.

### 3.5. Tgfb3 Deletion Leads to Hyper-Activated SMAD2/3 and SMAD1/5 Signaling Pathways

**Figure 6:**
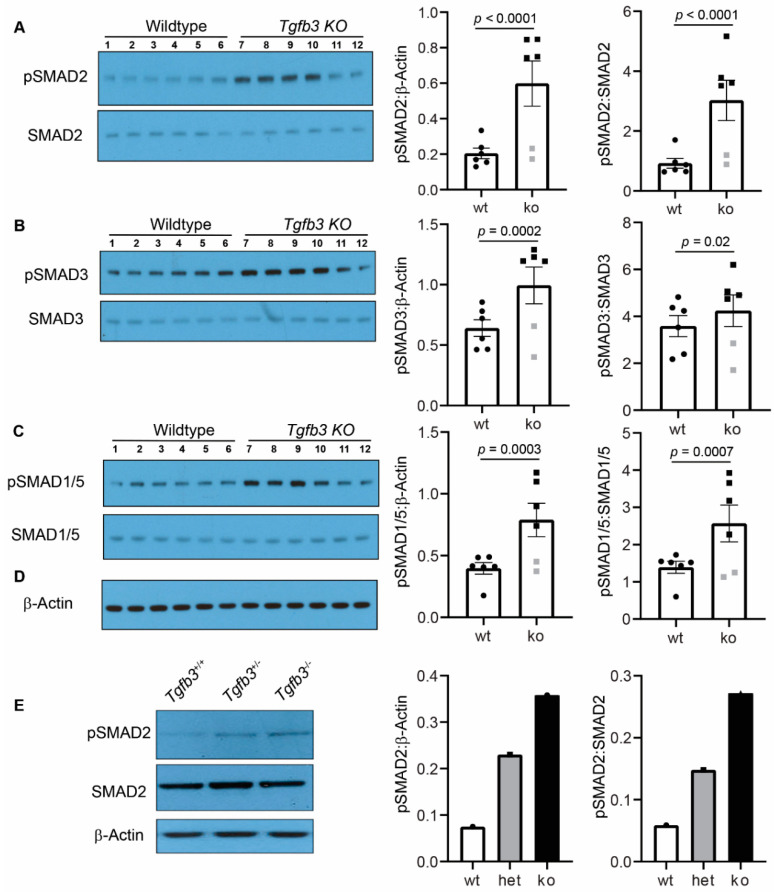
TGFβ3 is required for TGFβ-SMAD cardiovascular pathway activation. (**A**–**C**), Western blot analysis of fetal hearts and the accompanied ascending aorta (E18.5) shows protein levels of the phosphorylated SMADs (pSMAD2, pSMAD3, pSMAD1/5) and total SMADs (SMAD2, SMAD3, SMAD1/5). (**D**) A common and independent β-actin blot (**C**, bottom) was used for normalizing data from pSMAD2, pSMAD3, and pSMAD1/5 blots. There was no difference in the β-actin levels between any cardiovascular tissues taken from wildtype and Tgfb3^−/−^ mice. Densitometric quantification of phosphorylated proteins after normalization to β-actin and total non-phosphorylated proteins is shown on the right (**A**–**C**). E, Representative western blots of pooled samples (3 hearts and aortas/sample) from wildtype, *Tgfb3*^+/−^, and *Tgfb3*^−/−^ fetuses (E14.5) for pSMAD2, SMAD2, and β-actin. Each western blot was repeated three times with similar results. Western blots and densitometric quantification show the levels of pSMAD2 (60 kDa) and SMAD2 (60 kDa) (**A**,**D**), pSMAD3 (52 kDa) and SMAD3 (52 kDa) (**B**), pSMAD1/5 (60 kDa) and SMAD1/5 (60 kDa) (**C**), and β-actin (42 kDa) (**A**–**D**). Note that levels of pSMAD2, pSMAD3, and pSMAD1/5 are increased in individual samples taken from TGFβ3-deficient mice (**A**–**C**). The levels of pSMAD2 were also higher in pooled samples of both *Tgfb3*^+/−^ and *Tgfb3*^−/−^ fetuses compared to pooled sample from wildtype fetuses. *Tgfb3*^−/−^ had slightly higher levels of pSMAD2 than the *Tgfb3*^+/−^ fetuses. Data points excluded from statistical analysis (Student’s *t* test) are indicated by grey symbols (**A**–**C**). Individual densitometric values from each pooled samples were plotted (**E**). All western blots (**A**–**C**) with individual samples were done in triplicate. Thus, all data points represent an average values of three independent blots (**A**–**C**). *p*-values are shown in the figure. Numerical data from multiple individual samples are presented as scatter dot-plots with boxes denoting the mean; error bars indicate the SEM.

To determine the role of TGFβ3 in the regulation of downstream SMADs, the activation of important downstream TGFβ-dependent SMADs in isolated whole hearts with attached ascending aorta from the wildtype (*n* = 6) and *Tgfb3*^−/−^ (*n* = 6) fetuses (E18.5) was determined by Western blot analysis. The changes in the TGFβ receptor-dependent phosphorylation of serine/threonine residues of SMADs (i.e., pSMAD2), SMAD3 (i.e., pSMAD3), and SMAD1/5 (i.e., pSMAD1/5) were quantified via densitometric analysis from the Western blots. There was no significant change in the total amount of the SMAD2, SMAD3, SMAD1/5, and β-actin proteins in the *Tgfb3*^−/−^ hearts compared to wildtype hearts (Figure 6A–C). Importantly, these biochemical data showed that the levels of pSMAD2, pSMAD3, and pSMAD1/5 were significantly increased in the hearts of the *Tgfb3*^−/−^ fetuses compared to the wildtype fetuses (Figure 6A–C). We found that 2/3 cases of mutants fall within a high SMAD-dependent TGFβ signaling cluster, whereas the levels of activated SMADs were not significantly altered in one third of cases of *Tgfb3*^−/−^ fetuses compared to wildtype fetuses. Biochemical analysis of ‘pooled’ tissue samples (three hearts with ascending aortas/sample) confirmed elevated pSMAD2 in *Tgfb3*^−/−^ fetuses and also revealed increased pSMAD2 in *Tgfb3*^+/−^ fetuses compared to wildtype fetuses at E14.5 (Figure 6E). Importantly, pSMAD2 levels were higher in *Tgfb3*^−/−^ fetuses than the *Tgfb3*^+/−^ fetuses, suggesting that loss of *Tgfb3* in a dose-dependent fashion triggers SMAD2 activation. Taken together, heterozygous or homozygous loss-of-function of *Tgfb3* results in paradoxically increased levels of activated SMAD2, SMAD3, and SMAD1/5 in the fetal cardiovascular tissues.

### 3.6. TGFβ3-Deficiency Triggers Activation of Non-Canonical MAPK-Mediated TGFβ Signaling Pathways

**Figure 7:**
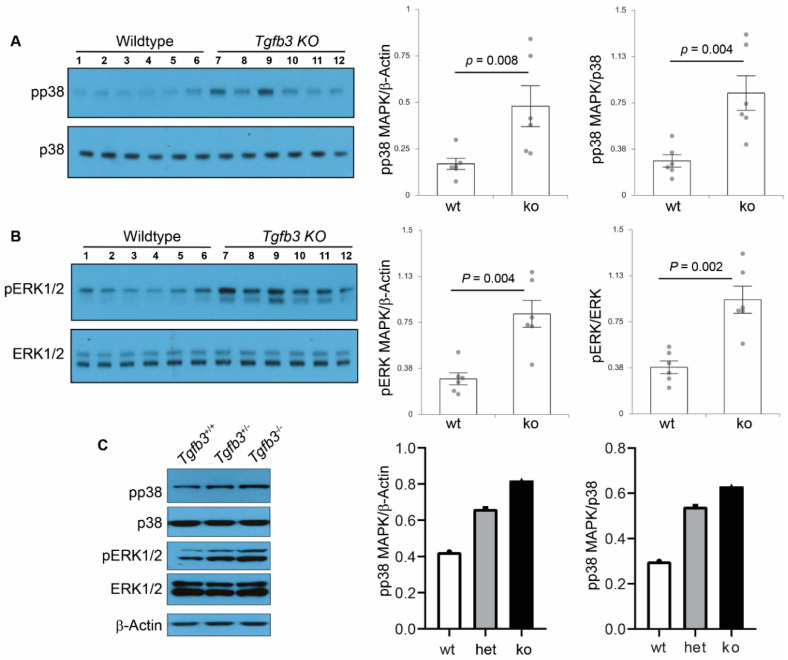
TGFβ3 is required for SMAD-independent non-canonical TGFβ cardiovascular pathway activation. (**A**,**B**) Western blot analysis of fetal hearts and accompanying ascending aortas (E18.5) show protein levels of phosphorylated p38 MAPK (pp38), total p38 MAPK (p38), phosphorylated ERK1/2 MAPK (pERK1/2), and total ERK1/2 MAPK (ERK1/2). Densitometric quantification of phosphorylated proteins after normalization to β-actin (via a common β-actin blot used in Figure 6D) and total non-phosphorylated proteins is shown on the right (**A**,**B**). Western blots and densitometric quantification show levels of pp38 (43 kDa) and p38 (40 kDa) (**A**), pERK1/2 (44/42 kDa) and ERK1/2 (44/42 kDa) (**A**,**B**). Note that similar results are obtained with normalization to either β-actin or the respective total p38 and ERK1/2 proteins. Note also that levels of pp38 MAPK and pERK1/2 MAPK are significantly increased in TGFβ3-deficient cardiovascular tissues. No data points were excluded from statistical analysis. Nonparametric test (Mann Whitney test) was used for analysis. *p*-values are shown in the figure. Numerical data are presented as scatter dot-plots with boxes denoting the mean; error bars indicate the SEM. (**C**) Representative western blots of pooled samples (3 hearts and aortas/sample for each genotype) showing elevated levels of pp38 and pERK1/2 in *Tgfb3*^+/−^ and *Tgfb3*^−/−^ fetuses (E14.5). Each western blot was repeated three times with similar results. Individual values for each samples were plotted. Notably, there is a trend of higher levels of both pp38 and pERK1/2 in *Tgfb3*^−/−^ fetal hearts compared to *Tgfb3*^+/−^ fetuses.

TGFβ signaling under pathophysiological conditions can occur via non-SMAD pathways, including p38 MAPK and ERK1/2 MAPK [23]. Consequently, Western blot analysis was done on wildtype (*n* = 6) and *Tgfb3*^−/−^ (*n* = 6) whole individual hearts with ascending aorta to determine the changes in the downstream components of the non-canonical TGFβ pathway. The densitometric analysis found no change in the levels of β-actin and total p38 protein (Figure 7A). Importantly, the data revealed significantly increased activation of p38 MAPK (i.e., pp38 MAPK) in the *Tgfb3*^−/−^ hearts and aorta compared to the wildtype (Figure 7A). Moreover, the data showed that levels of activated ERK1/2 MAPK (i.e., pERK1/2), when normalized to total ERK1/2, were also higher in the cardiovascular tissues of the *Tgfb3*^−/−^ fetuses than the wildtype (Figure 7B). Finally, Western blot analysis of pooled cardiovascular tissue samples (three hearts and aortas/sample) not only confirmed the elevated levels of pp38 and pERK1/2 in *Tgfb3*^−/−^ fetuses but they also revealed increased levels of pp38 and pERK1/2 in Tgfb3^+/−^ fetuses compared to wildtype fetuses (Figure 7C). Additionally, pp38 and pERK1/2 levels were higher in *Tgfb3*^−/−^ fetuses than the *Tgfb3*^+/−^ fetuses, suggesting that loss of *Tgfb3* in a dose-dependent fashion triggers the activation of both pp38 and pERK1/2 MAPKs. Collectively, these findings indicate that heterozygous or homozygous loss of *Tgfb3* in vivo leads to hyper-activation of SMAD-independent non-canonical TGFβ signaling through p38 MAPK and ERK1/2 MAPK pathways.

## 4. Discussion

This study has established that a significant fraction of the *Tgfb3*^−/−^ mice develop a variety of cardiovascular malformations including DORV, ventricular septal defects (OFT, perimembranous, muscular), abnormal vascular walls, aortic valve and AV valve thickening, and impaired ventricular myocardium; especially that of the RV. Approximately two thirds of cases of the *Tgfb3*^−/−^ mice develop one or more cardiovascular malformations and these phenotypic differences are not due to the genetic background of these mice since all mice used in this study are on the C57BL/6 background produced via non-sibs mating. In addition, *Tgfb3*^+/−^ fetuses develop mild cardiovascular malformations, including hypoplastic arterial walls, OFT cushion thickening, and impaired ventricular myocardium. These findings are consistent with the gene expression studies reported here and previous studies showing strong *Tgfb3* expression in the developing heart [26]. Two studies, in particular, established that systemic deletion of *Tgfb3* in mice leads to a cleft palate and perinatal lethality [1,2]. *Tgfb3*^−/−^ mice (one third of cases), which do not have any obvious cardiovascular structural defects, still die perinatally and have a cleft palate. *Tgfb3* heterozygous mice survive normally. Many mutations in mice that cause a cleft palate also lead to heart defects [37]. *TGFB3* mutations in the LDS5 (also known as RNHF syndrome) typically cause cleft palate and is also characterized by several cardiovascular malformations, including thoracic and/or abdominal aneurysms, persistent foramen ovale, atrial or ventricular septal defects, atrioventricular block, aortic and mitral valve disease, and familial arrhythmogenic right ventricular dysplasia (ARVD1/C) involving replacement of the ventricular myocardium with fatty and fibrous elements, preferentially in the free wall of the right ventricle [9,12,13,38]. It is interesting that *Tgfb3*^−/−^ fetuses have no atrial septal defects (ASD) and inflow tract abnormalities such as atrioventricular septal defects (AVSD), double inlet left ventricle (DILV), and common AV canal. This would indicate that abnormal OFT remodeling such as related to NCC and anterior SHF may be involved in OFT-related defects of *Tgfb3*^−/−^ embryos. Overall, the data presented in this study is consistent with clinical genetic findings and indicate that TGFβ3 is required for multiple important aspects of cardiovascular development.

*Tgfb3* is expressed in post-EMT cushion mesenchyme during cushion remodeling and differentiation, which is consistent with the OFT and AV cushion remodeling and OFT septation defects in *Tgfb3*^−/−^ embryos. This is a contrast with the *Tgfb2*^−/−^ embryos which develop both OFT and AV septation defects [39]. The data is also in line with previous studies indicating that TGFβ3 is not expressed in the mouse heart during EMT and that the cardiac EMT is not affected in *Tgfb3*^−/−^ embryos [40,41]. TGFβs are known to induce apoptosis [42,43,44]. As a consequence, the effect of absence of TGFβ3 might result in down-regulated apoptosis, as shown here. Therefore, it is unlikely that down-regulated TGFβ3 represses apoptosis. Furthermore, latent TGFβ3, as demonstrated in the SHF [45] needs to be activated and apoptotic neural crest cells, by changing the micro-environment, might just activate TGFβs. Definitive proof that this chain of events is reversed involves experiments inhibiting apoptosis by deleting, e.g., apoptosis-specific genes as performed by Watanabe and Fisher [46] in the chicken embryo, which included outflow tract malformations even though the focus was not on TGFβ signaling. In addition, cell lineage tracing superimposed on systemic or conditional *Tgfb3* null embryos will be another approach to reveal the effect of *Tgfb3* on cellular apoptosis in the OFT septum. Collectively, the OFT cushion remodeling defects are consistent with the presence of both aortic and pulmonary valve thickening in the *Tgfb3*^−/−^ fetuses. OFT septal malformations such as the DORV, are mainly subaortic in nature and exhibit defective fusion and remodeling of the outlet septum in *Tgfb3*^−/−^ embryos. In some of the *Tgfb3*^−/−^ fetuses, the aorta was positioned directly over a VSD due to an OFT alignment defect, which might be the result of abnormal inner curvature remodeling. On the other hand, the AV cushion remodeling defects are involved in the thickening of mitral and/or tricuspid valves in *Tgfb3*^−/−^ fetuses. Notably, both outflow tract cushion and AV cushion defects are not present in all *Tgfb3*^−/−^ fetuses. The overall cardiovascular tissue expression of *Tgfb3* is quite distinct but can slightly overlap to that of *Tgfb2* during cardiovascular development [26,47]. It is possible that TGFβ2 could slightly compensate for the loss of TGFβ3 during development. These suggestions are consistent with the clinical findings indicating the presence of aortic and mitral valve diseases and aortic aneurysm in the LDS patients with genetic mutations in *TGFB2* (LDS4) or TGFB3 (LDS5) [9,38,48]. Such a prediction is also supported by the observation that *Tgfb3* deletion worsens embryonic developmental phenotypes of *Tgfb2* knockout embryos [49]. Importantly, *Tgfb3* expression increases in comparison to *Tgfb2* during heart development [47]. This raises a possibility whether TGFβ3 could represent the predominant TGFβ isoform during later stages of cardiac remodeling, a suggestion consistent with the mutations in TGFB3 in adult cardiac disease patients [7,50].

*TGFB3* mutations result in dysregulated collagen matrix reorganization and fibrotic lesions associated with the ventricular fibrosis and aortic or mitral valve disease [6]. Several studies have indicated that TGFβ signaling is required for collagen production, remodeling, and organization [35,51,52]. Collagen organization is critical for heart development, and structure and function in the adult myocardium and heart valves [28]. A direct role of TGFβ3 on collagen reorganization has determined for the first time in this study. The data reveals that TGFβ3-deficient whole mouse fibroblasts are defective in their ability to reorganize the collagen matrix and that exogenous addition of higher doses of TGFβ1 can compensate for the loss of TGFβ3. This supports the hypothesis that loss of *Tgfb3* results in a compensatory TGFβ ECM response. This failure of reorganization is indicative of altered contractile machinery and/or adhesion molecules [33]. *TGFB3* mutations have also been reported in patients with mitral and aortic valve disease and that hyper-activated TGFβ signaling has been implicated in mammalian valve disease [9,53]. Thus, the results presented in this study, establishing a unique and important requirement of TGFβ3 in collagen fiber organization, are novel and represent a significant step which will increase our understanding of the role of TGFβ3 signaling in heart development and cardiac pathophysiology.

One of the observations and potential limitations of our study is the variable phenotypic penetrance of cardiovascular anomalies in *Tgfb3*^−/−^ mice, which hinders the morphometric comparison and the investigation of the underlying cellular, developmental, molecular and biochemical mechanisms. Given that pSMAD2/3 levels are not significantly increased in one third of cases of the mutant hearts, it is difficult to conclude from the results whether the increased pSMAD2/3 levels cause the cardiovascular malformations in the *Tgfb3*^−/−^ fetuses. It is possible that increased TGFβ signaling instead represents a compensatory mechanism and that a failure to fully compensate for the loss of TGFβ3 signaling results in phenotypically variable cardiovascular anomalies in the *Tgfb3*^−/−^ fetuses. Such a prediction is consistent with more severe developmental phenotype and mid-gestational lethality reported in *Tgfb3* and *Tgfb2* double knockout embryos compared to the cardiovascular phenotypes reported in the *Tgfb3*^−/−^ embryos in our study [49].

Another very significant finding in this study is that genetic disruption of *Tgfb3* leads to impaired ventricular myocardium. Regulatory mutations in the *TGFB3* associated with the increased TGFβ signaling have been identified in patients showing a typical ARVD1/C phenotype [7]. Notably, fibro-fatty replacement of the right ventricular myocardium in these ARVD1/C patients involves both fibrosis and myocardial apoptosis [6]. Our data suggest that TGFβ3-deficient hearts exhibit increased activation of SMAD-dependent (SMAD2/3 and SMAD1/5) and non-SMAD (p38 MAPK and ERK1/2 MAPK) pathways. TGFβ signaling via the SMAD2/3 pathways can induce the expression of extracellular matrix genes and the phenotypic transformation of fibroblasts into myofibroblasts, and stimulate pro-collagen and collagen synthesis [54]. Moreover, increased TGFβ signaling via the TRAF6-TAK1-p38 MAPK pathway can lead to apoptosis. In a remarkable study, Li et al. (2011) have shown that the loss of Jup1 (i.e., plakoglobin), which leads to impaired cell differentiation, increased myocardial apoptosis and fibrosis, and elevated TGFβ signaling causes ARVD1/C in mice [8]. Mutations in genes encoding desmosomal proteins including plakoglobin (JUP1) have also been reported in about half of the ARVC patients. Thus, it is possible that increased TGFβ signaling is a major and common underlying mechanism of ARVC pathogenesis. We provided histological and biochemical evidence showing that mild cardiovascular malformations (such as hypoplastic arterial walls, myocardial defects, OFT cushion defects) in Tgfb3^+/−^ embryos are also associated with hyper-activated canonical and noncanonical TGFβ signaling. This observation is quite similar to the increased SMAD2 activation seen in aortic tissue and fibroblasts of LDS5 and ARVD1/C patients harboring *TGFB3* heterozygous mutations. Together, this suggests that increased TGFβ signaling may be a compensatory response to the loss of TGFβ3 and that the extent of TGFβ overactivation beyond a certain threshold (i.e., wildtype < *Tgfb3* heterozygous < *Tgfb3* homozygous) could result in range of cardiovascular pathomorphologies. Since this increased TGFβ signaling seems compatible with the survival of *Tgfb3*^+/−^ mice, it is possible that adult *Tgfb3*^+/−^ mice will develop progressive cardiovascular disease such as aortic aneurysm, valvular heart disease, and cardiomyopathy. We are currently analyzing adult *Tgfb3*^+/−^ mice at different ages in longitudinal studies to establish a correlation between TGFβ hyperactivation and development and progression of cardiovascular pathophysiology and disease (i.e., cardiac dysfunction, heart valve disease, aortic aneurysm via echocardiography).

In this study, we describe the abnormal vascular walls (aorta and pulmonary trunk) phenotype in both *Tgfb3* heterozygous and *Tgfb3* knockout fetuses, which nicely links this phenotype to LDS5 (human *TGFB3* mutations). Our data also show the expression of *Tgfb3* in the great vessels as well as in the condensed mesenchyme of the OFT septum, which is consistent with earlier findings [26]. That is a rare combination. Since, in the OFT (vascular and septation), the NCC interact with SHF [55,56,57], the total phenotypic outcomes in *Tgfb3* mutants may be the effect of a lack of TGFβ3 on both cell types. Since adventitial fibroblasts receive arterial epicardial derived cells [58], it is possible that the epicardial cells in the abnormal vascular walls in *Tgfb3*^−/−^ embryos could be defective. The myocardial defects described in this study may be based on a defective contribution of epicardium derived fibroblasts with a higher involvement (but not selective) of the RV. The atrioventricular valves, both mitral and tricuspid receive epicardial cells [58,59,60], and therefore TGFβ3 might play an important role in epicardial morphogenesis. *Tgfb3* expression data support the idea that the role of epicardium is a crucial link to ventricular myocardial problems and the AV cushions [26,60]. Both ventricular myocardium and AV cushions receive epicardial cells that mainly differentiate into fibroblasts [58,59,60]. There is a difference in homing and timing of epicardium to RV and LV [61], which could explain why, in *Tgfb3*^−/−^ embryos, the RV is more involved, although surely the LV is also affected as well. There are several models in which epicardium is defective, either after mechanical removing the epicardium in the quail or in mouse mutant models (PDGFRα, Podoplanin, RxR) [58,62]. All of these animal models present with very thin myocardium and a spongy ventricular septum with often muscular VSD. Recently, *Tgfb3* conditional knockout mice have been generated [63] and will be useful in future investigation to determine the cell-specific role of TGFβ3 in cardiovascular development and adult cardiovascular disease. In conclusion, TGFβ3 is required for cardiovascular development in mice by maintaining a proper balance of downstream SMAD-dependent and MAPK-dependent TGFβ signaling mechanisms.

## Figures and Tables

**Table 1:** Summary of cases with or without cardiovascular defects in *Tgfb3* knockout mice (Embryonic Day 13.5 to 18.5; *n* = 19).

ID#	Genotype	Age	DORV	AV Thickening	PV Thickening	Vascular walls Abnormalities	VSD	AVSD	Thin Myocardium	RV Hyperplasia	MV Thickening	TV Thickening
KO1	*Tgfb3* ^−/−^	e16.5-17.5	No	No	No	No	No	No	No	No	No	No
KO2	*Tgfb3* ^−/−^	e13.5	No	Yes	Yes	No	Perimembranous	No	Yes	No	Yes	Yes
KO3	*Tgfb3* ^−/−^	e14.5-15.5	No	No	No	No	No	No	No	No	Yes	Yes
KO4	*Tgfb3* ^−/−^	e14.5-15.5	No	Yes	Yes	No	Muscular	No	No	No	Yes	Yes
KO5	*Tgfb3* ^−/−^	e14.5-15.5	No	Yes	Yes	No	Muscular	No	Yes	No	No	No
KO6	*Tgfb3* ^−/−^	e14.5-15.5	No	No	No	No	No	No	No	No	No	No
KO7	*Tgfb3* ^−/−^	e14.5-15.5	No	No	No	No	No	No	No	No	No	No
KO8	*Tgfb3* ^−/−^	e16.5-17.5	No	No	No	No	No	No	No	No	No	No
KO9	*Tgfb3* ^−/−^	e13.5	No	Yes	Yes	No	Perimembranous	No	No	Yes	Yes	Yes
KO10	*Tgfb3* ^−/−^	e16.5-17.5	No	Yes	Yes	No	No	No	No	No	No	No
KO11	*Tgfb3* ^−/−^	e14.5-15.5	No	Yes	Yes	ND	No	No	No	Yes	Yes	Yes
KO12	*Tgfb3* ^−/−^	e18.5	No	Yes	Yes	Yes	No	No	Yes	No	No	No
KO13	*Tgfb3* ^−/−^	e18.5	No	Yes	Yes	Yes	Perimembranous	No	No	Yes	No	No
KO14	*Tgfb3* ^−/−^	e15.5-16.5	No	Yes	Yes	No	No	No	Yes	No	No	No
KO15	*Tgfb3* ^−/−^	e15.5-16.5	Subaortic	Yes	Yes	Yes	Perimembranous	No	No	Yes	Yes	Yes
KO16	*Tgfb3* ^−/−^	E18.5	No	No	No	No	No	No	No	No	No	No
KO17	*Tgfb3* ^−/−^	e13.5	No	Yes	Yes	No	No	No	No	No	Yes	Yes
KO18	*Tgfb3* ^−/−^	e14.5	No	No	No	No	No	No	No	No	No	No
KO19	*Tgfb3* ^−/−^	e13.5	No	Yes	Yes	No	Muscular	No	Yes	No	Yes	Yes
	Number of cases (%)		1 (5.2%)	12 (63.15%)	12 (63.15%)	3 (15.7%)	P: 4 (21%); M: 3 (15.7%)	0 (0%)	6 (31.5%)	4 (21%)	8 (42.1%)	8 (42.1%)
Total cases	*Tgfb3* ^−/−^	19										
Total cases	Wildtype *(Tgfb3^+/+^)*	14										

Abbreviations: DORV, double outlet right ventricle; AV, atrioventricular valve; PV, pulmonary valve; VSD, ventricular septal defect; AVSD, atrioventricular septal defect; RV, right ventricule; MV, mitral valve; TV, tricuspid valve.

## Supplementary Materials

The following are available online at https://www.mdpi.com/2308-3425/7/2/19/s1. Table S1. Cardiovascular defects in *Tgfb3* knockout mice (Embryonic Day 13.5 to 18.5 (n = 19). Figure S1. Systemic *Tgfb3* deletion disrupts ventricular myocardial development and leads to muscular VSD. A–C, H&E stained sections of wildtype and different *Tgfb3*^−/−^ fetuses (E15.5-16.5) showing abnormal size, shape, and myocardium of the right ventricle in *Tgfb3*^−/−^ (B,C, left arrow) and mitral valve thickening (B,C, arrowheads). The left ventricular myocardium in *Tgfb3*^−/−^ fetuses (B,C, right arrow) was also not normal. D,E, Cardiac muscle actin (clone HHF35) immunohistochemistry of cross sections of E14.5-15.5 fetuses showing myocardium of both right and left ventricles was affected in some *Tgfb3*^−/−^ resulting in muscular VSD (E, arrow). F,G, H&E stained sections of wildtype and *Tgfb3*^+/−^ fetuses (E14.5-15.5) showing mild thinning of the right ventricular myocardium (G, left arrowhead) and moderately thickened left ventricular myocardium (G, right arrowhead) in *Tgfb3*^+/−^ fetuses compared to wildtype heart (F). Scale bars: 200 µm for A–G. Abbreviations: rv, right ventricle; lv, left ventricle; tv, tricuspid valve; mv, mitral valve. Figure S2. *Tgfb3* deletion leads to pulmonary and aortic valve defects. A–D, Hematoxylin and eosin staining for E15.5 wildtype (A,D), *Tgfb3^+/−^* (B,D), and *Tgfb3*^−/−^ (C,F) fetuses. *Tgfb3^+/−^* fetus displays thinning of vascular walls of aorta and pulmonary trunk (B, arrowheads), mild thickening of both pulmonary (B, arrow) and aortic (E, arrow) valves compared to wildtype fetus (A,B). Notably, *Tgfb3*^−/−^ fetuses develop severe forms of these cardiovascular defects (C,F). Scale bars: 200 µm for A-F. Figure S3. Abnormal ascending aortic walls in *Tgfb3* knockout fetuses. A–D, Elastin autofluorescence (C,D) of hematoxylin and eosin-stained (A,B) sections. Compared to wildtype littermate (C), *Tgfb3*^−/−^ fetus shows poorly formed elastic lamellae and disorganized vascular smooth muscle cells in the aortic wall (arrows, D). Fluorescence images (C,D) were taken from region of aorta indicated by boxes (A,B). Arrows indicate elastic fibers (C,D) and the white dotted lines demarcates the aortic wall from vaso vasorum (D). Scale bars = 100 µm for A,B; 25 µm for C,D. Figure S4. Measurement of aortic valve volume in *Tgfb3* knockout fetuses. A–G, Morphometric comparison and volume measurements using AMIRA 3D segmentation of aortic valves from wildtype (A,C,E) and *Tgfb3*^−/−^ (B,D,F) embryos (E15.5) showing non-coronary leaflets in red, left coronary leaflets in green, and the right coronary leaflets in yellow. The hyperplastic nature of the outflow tract cushions in *Tgfb3*^−/−^ embryos compared to the wildtype littermate embryos (G). Student’s *t* test was used. *p*-Values are indicated in the histogram. Numerical data are presented as scatter dot-plots with boxes, with the box denoting the mean; error bars identify the S.E.M (n = 3 per genotype).
