# Supplementary material for: Transforming Growth Factor Beta3 is Required for Cardiovascular Development
Source: J Cardiovasc Dev Dis. 2020 May 24;7(2):19. doi: 10.3390/jcdd7020019 (PMC7344558; doi:10.3390/jcdd7020019)
Supplement: Supplementary file 1 [file jcdd-07-00019-s001.pdf]

Journal of
Cardiovascular Development and Disease

MDPI

Supplementary Materials

Transforming growth factor beta3 is required for cardiovascular development

Mrinmay Chakrabarti [1], Nadia Al-Sammarraie [1], Mengistu G. Gebere [1], Aniket Bhattacharya [1], Sunita Chopra [1], John Johnson [1], Edsel A. Peña [2], John F. Eberth [1], Robert E. Poelmann [3], Adriana C. Gittenberger-de Groot [3] and Mohamad Azhar [1,4,*]

1 Department of Cell Biology and Anatomy, University of South Carolina School of Medicine, Columbia, SC 29209; Mrinmay.Chakrabarti@uscmed.sc.edu (M.C.); Nadia.Al-Sammarraie@uscmed.sc.edu (N.A.-S.); Mengistu.Gebere@uscmed.sc.edu (M.G.G.); Aniket.Bhattacharya@uscmed.sc.edu (A.B.); sunita.chopra42@gmail.com (S.C.); John.Johnson@uscmed.sc.edu (J.J.), John.Eberth@uscmed.sc.edu (J.F.E.)
2 Department of Statistics, University of South Carolina, Columbia, SC 290208; pena@stat.sc.edu
3 Department of Cardiology, Leiden University Medical Center, Leiden, The Netherlands; R.E.Poelmann@lumc.nl (R.E.P.); A.C.Gittenberger-de_Groot@lumc.nl (A.C.G.d.-G.)
4 William Jennings Bryan Dorn VA Medical Center, Columbia, SC 29209
*Correspondence to: Mohamad.Azhar@uscmed.sc.edu

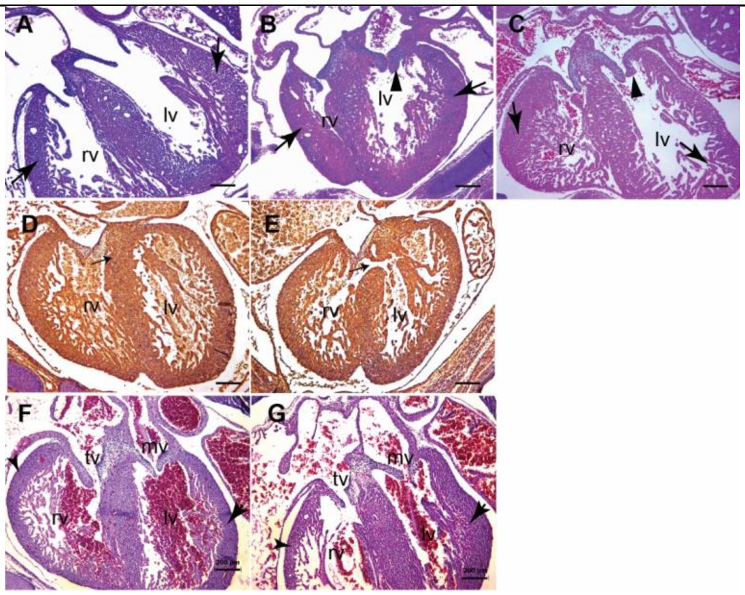

Figure S1. Systemic *Tgfb3* deletion disrupts ventricular myocardial development and leads to muscular VSD. **A–C,** H&E stained sections of wildtype and different *Tgfb3*$^{-/-}$ fetuses (E15.5-16.5) showing abnormal size, shape, and myocardium of the right ventricle in *Tgfb3*$^{-/-}$ (**B**,**C**, left arrow) and mitral valve thickening (**B–C**, arrowheads). The left ventricular myocardium in *Tgfb3*$^{-/-}$ fetuses (B-C, right arrow) was also not normal. **D–E,** Cardiac muscle actin (clone HHF35) immunohistochemistry of cross sections of E14.5-15.5 fetuses showing myocardium of both right and left ventricles was affected in some *Tgfb3*$^{-/-}$ resulting in muscular VSD (**E,** arrow). **F–G,** H&E stained sections of wildtype and *Tgfb3*$^{+/-}$ fetuses (E14.5-15.5) showing mild thinning of the right ventricular myocardium (**G,** left arrowhead) and moderately thickened left ventricular myocardium (**G**, right arrowhead) in *Tgfb3*$^{+/-}$ fetuses compared to wildtype heart (**F**). Scale bars: 200 μm for **A–G**. Abbreviations: rv, right ventride; lv, left ventricle; tv, tricuspid valve; mv, mitral valve.

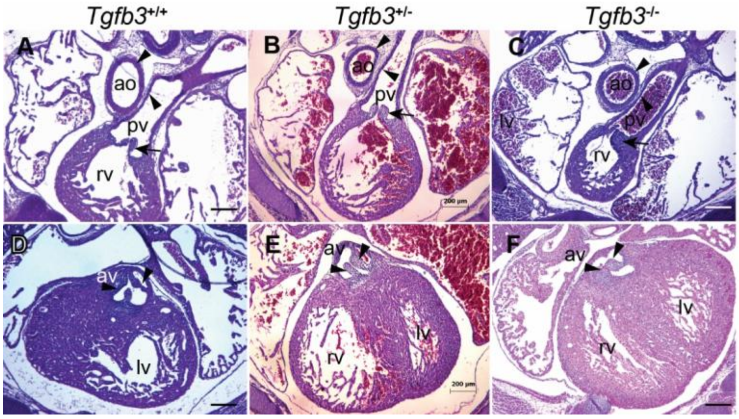

Figure S2. *Tgfb3* deletion leads to pulmonary and aortic valve defects. **A–D,** Hematoxylin and eosin staining for E15.5 wildtype (**A**,**D**), *Tgfb3*$^{+/-}$ (**B**,**D**), and *Tgfb3*$^{-/-}$ (**C**,**F**) fetuses. *Tgfb*$^{+/-}$ fetus displays thinning of vascular walls of aorta and pulmonary trunk (**B**, arrowheads), mild thickening of both pulmonary (**B**, arrow) and aortic (**E**, arrow) valves compared to wildtype fetus (**A**,**B**). Notably, *Tgfb3*$^{-/-}$ fetuses develop severe forms of these cardiovascular defects (**C**,**F**). Scale bars: 200 μm for **A–F**.

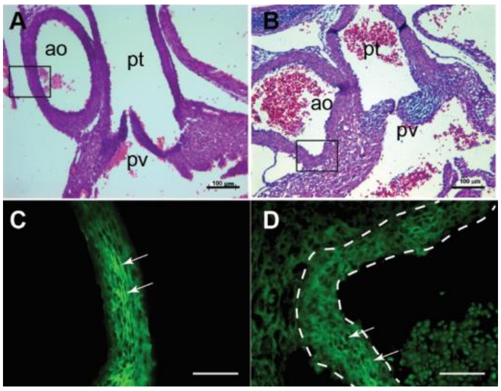

Figure S3. Abnormal ascending aortic walls in *Tgfb3* knockout fetuses. **A-D,** Elastin autofluorescence (**C**,**D**) of hematoxylin and eosin-stained (**A**,**B**) sections. Compared to wildtype littermate (**C**), *Tgfb3*$^{-/-}$ fetus shows poorly formed elastic lamellae and disorganized vascular smooth muscle cells in the aortic wall (arrows, **D**). Fluorescence images (**C**,**D**) were taken from region of aorta indicated by boxes (**A**,**B**). Arrows indicate elastic fibers (**C**,**D**) and the white dotted lines demarcates the aortic wall from vaso vasorum (**D**). Scale bars = 100 μm for **A–B**; 25 μm for **C–D**.

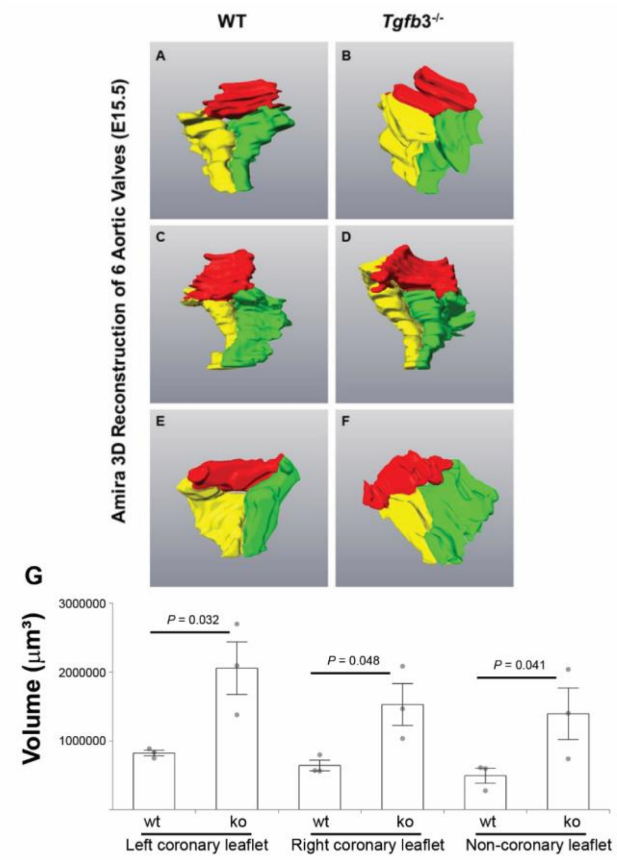

Figure S4. Measurement of aortic valve volume in *Tgfb3* knockout fetuses. **A–G,** Morphometric comparison and volume measurements using AMIRA 3D segmentation of aortic valves from wildtype (**A**,**C**,**E**) and *Tgfb3*$^{-/-}$ (**B**,**D**,**F**) embryos (E15.5) showing non-coronary leaflets in red, left coronary leaflets in green, and the right coronary leaflets in yellow. The hyperplastic nature of the outflow tract cushions in *Tgfb3*$^{-/-}$ embryos compared to the wildtype littermate embryos (**G**). Student's *t* test was used. *p*-values are indicated in the histogram. Numerical data are presented as scatter dot-plots with boxes, with the box denoting the mean; error bars identify the S.E.M (n = 3 per genotype).

Journal of
Cardiovascular Development and Disease

MDPI

Table S1. Cardiovascular defects in *Tgfb3* knockout mice (Embryonic Day 13.5–18.5 (n = 19).

**Abnormal Part; Type of Abnormality**	**No. of Cases**	**% of Cases**	**No. of Cases affected, Summary**	**% of Cases, Summary**
Outflow tract			12	63.15
Vascular walls abnormalities	3	15.7		
Thickening of PV±AoV	12	63.15		
DORV	1	5.2		
Septal defects			7	36.8
OFT malalignment and perimembranous (DORV±VSD)	4	21		
Muscular VSD	3	15.7		
AV valve			8	
Thickening of TV±MV	8	42.1		42.1
Ventricular myocardium			9	47.3
Hypoplasia compact/trabecular				
RV	5	26.3		
LV	5	26.3		
RV/LV	5	26.3	26.3	
Hyperplasia				
RV	4	21	21	
LV	4	21		
RV/LV	4	21		
No abnormality	6	31.5		31.5

Commented [MA2R1]: I re-formatted the table to fit in on a page and minimized multiple spaces

Commented [M1]: Please reformat the table (avoid using multiple spaces)
